# CNV-Finder: Streamlining Copy Number Variation Discovery

**DOI:** 10.1101/2024.11.22.624040

**Published:** 2024-11-23

**Authors:** Nicole Kuznetsov, Kensuke Daida, Mary B. Makarious, Bashayer Al-Mubarak, Kajsa Atterling Brolin, Laksh Malik, Cedric Kouam, Breeana Baker, Miriam Ostrozovicova, Katherine M. Andersh, Pin-Jui Kung, Yasser Mecheri, Yi-Wen Tay, Behloul Soundous Malek, Nada Al Tassan, Maria Teresa Periñan, Samantha Hong, Mathew Koretsky, Lana Sargeant, Kristin Levine, Cornelis Blauwendraat, Kimberley J. Billingsley, Sara Bandres-Ciga, Hampton L. Leonard, Huw R. Morris, Andrew B. Singleton, Mike A. Nalls, Dan Vitale, The Global Parkinson’s Genetics Program (GP2)

**Affiliations:** 1. Center for Alzheimer’s and Related Dementias (CARD), National Institute on Aging and National Institute of Neurological Disorders and Stroke, National Institutes of Health, Bethesda, MD, USA.; 2. DataTecnica LLC, Washington, DC 20037, USA; 3. Department of Clinical and Movement Neurosciences, Queen Square Institute of Neurology, University College London, London, UK; 4. King Faisal Specialist Hospital and Research Center, Riyadh, Saudi Arabia; 5. Translational Neurogenetics Unit, Department of Experimental Medical Science, Lund University, Lund, Sweden; 6. Centre for Preventive Neurology, Wolfson Institute of Population Health, Queen Mary University of London, London, UK; 7. Department of Neurology, P.J. Safarik University, Kosice, Slovak Republic; 8. Department of Neurology, University Hospital of L. Pasteur, Kosice, Slovak Republic; 9. Department of Neuromuscular Diseases, UCL Queen Square Institute of Neurology, London, UK; 10. Genome and Systems Biology Degree Program, National Taiwan University and Academia Sinica, Taipei, Taiwan; Division of Plastic Surgery, Department of Surgery, National Taiwan University Hospital, Taiwan; 11. Neurology Department, Dr Benbadis University Hospital, Constantine, Algeria; 12. University of Malaya, Kuala Lumpur, Malaysia; 13. Unidad de Trastornos del Movimiento, Servicio de Neurología y Neurofisiología Clínica, Instituto de Biomedicina de Sevilla, Hospital Universitario Virgen del Rocío/CSIC/Universidad de Sevilla, Seville, Spain; 14. Laboratory of Neurogenetics, National Institute on Aging, National Institutes of Health, Bethesda, MD 20892, USA; 15. School of Nursing, Virginia Commonwealth University, Richmond, VA, USA; 16. German Center for Neurodegenerative Diseases (DZNE), Tübingen, Germany; 17. Centre for Genetic Epidemiology, Institute for Clinical Epidemiology and Applied Biometry, University of Tübingen, Tübingen, Germany

**Keywords:** Python, Copy Number Variation (CNV), Structural Variant (SV), deep learning, long short-term memory (LSTM), genetics, pipeline

## Abstract

Copy Number Variations (CNVs) play pivotal roles in the etiology of complex diseases and are variable across diverse populations. Understanding the association between CNVs and disease susceptibility is of significant importance in disease genetics research and often requires analysis of large sample sizes. One of the most cost-effective and scalable methods for detecting CNVs is based on normalized signal intensity values, such as Log R Ratio (LRR) and B Allele Frequency (BAF), from Illumina genotyping arrays. In this study, we present CNV-Finder, a novel pipeline integrating deep learning techniques on array data, specifically a Long Short-Term Memory (LSTM) network, to expedite the large-scale identification of CNVs within predefined genomic regions. This facilitates the efficient prioritization of samples for subsequent, costly analyses such as short-read and long-read whole genome sequencing. We focus on five genes—Parkin (*PRKN*), Leucine Rich Repeat And Ig Domain Containing 2 (*LINGO2*), Microtubule Associated Protein Tau (*MAPT*), alpha-Synuclein (*SNCA*), and Amyloid Beta Precursor Protein (*APP*)—which may be relevant to neurological diseases such as Alzheimer’s disease (AD), Parkinson’s disease (PD), or related disorders such as essential tremor (ET). By training our models on expert-annotated samples and validating them across diverse cohorts, including those from the Global Parkinson’s Genetics Program (GP2) and additional dementia-specific databases, we demonstrate the efficacy of CNV-Finder in accurately detecting deletions and duplications. Our pipeline outputs app-compatible files for visualization within CNV-Finder’s interactive web application. This interface enables researchers to review predictions and filter displayed samples by model prediction values, LRR range, and variant count in order to explore or confirm results. Our pipeline integrates this human feedback to enhance model performance and reduce false positive rates. Through a series of comprehensive analyses and validations using both short-read and long-read sequencing data, we demonstrate the robustness and adaptability of CNV-Finder in identifying CNVs with regions of varied sparsity, noise, and size. Our findings highlight the significance of contextual understanding and human expertise in enhancing the precision of CNV identification, particularly in complex genomic regions like 17q21.31. The CNV-Finder pipeline is a scalable, publicly available resource for the scientific community, available on GitHub (https://github.com/GP2code/CNV-Finder; DOI 10.5281/zenodo.14182563). CNV-Finder not only expedites accurate candidate identification but also significantly reduces the manual workload for researchers, enabling future targeted validation and downstream analyses in regions or phenotypes of interest.

## Introduction

### Copy Number Variation Detection in Disease Genetics

Copy Number Variations (CNVs) are structural variants (SVs) that play key roles in the diversity of genetic architecture and disease risk. Spanning hundreds to hundreds of thousands of base pairs, these genomic variations, including duplications, deletions, insertions, and complex rearrangements, deviate from the expected number of copies within DNA segments^1^. Given the growing interest in understanding the contribution of SVs to disease heritability and susceptibility, these variants are examined across multiple genetic modalities, including genotyping arrays, short-read, and long-read whole genome sequencing (WGS). Among these approaches, genotyping arrays emerge as the most cost-effective and widely accessible method for large-scale investigations of genetic data. CNV detection is facilitated by leveraging the normalized signal intensity metrics derived from these assays, such as Log R Ratio (LRR) and B Allele Frequency (BAF)^1, 2, 3^. Enhancing the sensitivity and mitigating false positive rates of an array-based SV calling algorithm can expedite the identification of accurate candidates by significantly reducing the sample size requiring laborious human validation. Here we present CNV-Finder, a tool that enables targeted screening with genotyping arrays, effectively pinpointing regions for future investigation via more specialized, expensive methods such as long-read sequencing.

### Genes of Interest

We selected regions of interest for model development, surrounding suspected neurodegenerative disease (NDD) risk loci with 250 kilobase (kb) buffers, including Parkin (*PRKN*; chr6:161347557–162727802 [hg38]), Leucine Rich Repeat And Ig Domain Containing 2 (*LINGO2*; chr9:27948085–29213000 [hg38]), and Microtubule Associated Protein Tau (*MAPT*; chr17:45894381–46028333 [hg38]). These intervals exhibited sufficiently high rates of deletions and duplications to allow for the creation of large, robust training sets for both CNV types. The variants in these regions were well-defined, minimizing noise and ensuring consistency among manual evaluators involved in the classification of variants. Amyloid Beta Precursor Protein (*APP*; chr21:25880549–26171128 [hg38]) and alpha-Synuclein (*SNCA*; chr4:89724098–89838296 [hg38]) were included to demonstrate the final models’ usability on previously unseen genes with markedly lower frequencies. A buffer of 1 million base pairs was used to flank *APP* to increase the observed area and improve variant incidence, while a buffer of 3 million base pairs was used to better capture the scale of *SNCA’*s large SVs.

*PRKN* structural variants can cause autosomal recessive Parkinson’s disease (PD). However, the significance of common single heterozygous duplications and deletions in PD risk remains uncertain due to limited population-scale studies, resulting in conflicting findings^4, 5, 6^. Previous literature has also indicated potential disease-association between *LINGO2* SVs and conditions such as essential tremor (ET) and PD, with similar limitations due to sample size^7, 8^. Additionally, *MAPT* represents a particularly complex genomic region characterized by high linkage disequilibrium, distinct functional haplotypes, and a common inversion observed among Europeans^9^. This gene, along with neighboring genes such as *KANSL1* in chromosomal band 17q21.31, has attracted interest for potential associations with diseases including Progressive Supranuclear Palsy (PSP), Frontotemporal Dementia (FTD), Dementia with Lewy Bodies (DLB), and Alzheimer’s disease (AD)^10, 11, 12^. Previous literature has supported an association between the H1-haplotype of *MAPT* with additional diseases such as PD and other tauopathies like Corticobasal Degeneration (CBD), which may present clinically as Corticobasal Syndrome (CBS)^13, 14^. The *MAPT* H2 haplotype, on the other hand, has been associated with reduced risk in AD and explored for potential associations with other diseases^15, 16, 17^. Both *APP* and *SNCA* have strong evidence linking the genes to NDDs. Triplications and duplications in *SNCA* have been primarily linked to monogenic PD^18, 19, 20^. Mutations in *APP*, on the other hand, have strong implications in early-onset AD and cerebral amyloid angiopathy^21^. While *APP* deletions are less frequently studied, ancestry-specific evidence has indicated the existence of a Swedish *APP* deletion that is disease-causing^22, 23,24^. Utilizing the scalability of our pipeline, we developed a publicly available resource for the scientific community to conduct large-scale, parallelized searches across multiple cohorts to further investigate the role of SVs in the genetic architecture of NDDs, across wide ranges of phenotypes and ancestries.

## Materials and Methods

### Genotyping Data Inputs

All samples featured in both model training and testing underwent sequencing using the Illumina NeuroBooster array (NBA)^25^ and subsequent processing by the Global Parkinson’s Genetics Program (GP2)^26^. The NBA is specifically optimized for NDDs, therefore improving coverage in the regions we are analyzing and enhancing reliability in signal intensity measures. Notably, around 1.9 million variants are included in the array, with over 95,000 variants specific to the context of neurological disorders. One file containing the position, chromosome, BAF, and LRR values for all single nucleotide-polymorphism (SNPs) serves as the necessary input for the pipeline. To enhance data quality, users have the option to specify a customizable GenTrain (https://www.illumina.com/content/dam/illumina/gcs/assembled-assets/marketing-literature/gentrain-tech-note-m-gl-01258/gentrain-tech-note-m-gl-01258.pdf)^27^ score cut-off, which defaults to 0.2, thus filtering out SNPs with suboptimal calling quality. To observe the full range of potential predictions, no additional quality control was applied to the samples in our analysis; however, users may additionally upload a PLINK v1.9 (RRID:SCR_001757)^28^ bim format file or PLINK v2.3^29^ pvar format file to limit samples and variants to those that passed quality control detailed elsewhere^30^. Furthermore, users are required to specify either a gene name or chromosome, along with start and stop positions in base pairs, delineating the region of interest. All graphical representations and positional references in this paper adhere to the Genome Reference Consortium Human Build 38 (hg38)^31^.

### Samples

All GP2 cohorts involved in model creation and evaluation are delineated in Supplementary Table 1 and can be searched on GP2’s Cohort Dashboard by their study abbreviations (https://gp2.org/cohort-dashboard-advanced/). Supplementary Table 2 presents the manually curated set of 184 samples, spanning 9 ancestries with 156 cases and 28 controls, that were used in training preliminary models for each CNV type. This array data underwent visual assessment by a team of 13 NDD research scientists to identify samples exhibiting deletions or duplications of any size within *PRKN*. The preliminary training set comprises 70 deletions (Supplementary Table 2a), 29 duplications (Supplementary Table 2b), and 85 samples lacking visible CNVs in *PRKN* (Supplementary Table 2c). Short-read WGS was used to confirm the absence of visible structural variants in negative samples. The Accelerating Medicines Partnership program for Parkinson’s disease (AMP-PD) short-read WGS data collection has been previously outlined by Iwaki et al^32^ with additional details on SNV calling by Billingsley et al^33^. The Coriell Institute for Medical Research (CORIELL) samples with CNVs spanning various sizes and coverage levels, were withheld for subsequent validation of model performance and are summarized in Supplementary Table 3.

Cohorts chosen for model development, described in Table 1, encompass a broader spectrum of phenotypes to diversify the samples included in our training sets. The CORIELL and Parkinson’s Progression Markers Initiative (PPMI) cohorts are highlighted for their additional data availability, facilitating enhanced validation of predictions. While the former includes expert annotations held out for validation, the latter provides additional short and long-read WGS data for a subset of samples outlined in Supplementary Table 4. An additional subset of samples listed in Supplementary Table 5 cover more dementia-related phenotypes and were used for an in-depth exploration of the 17q21.31 region that encompasses *MAPT*.

### Long-read WGS

DNA was extracted from frozen blood samples as detailed in the protocol available at dx.doi.org/10.17504/protocols.io.x54v9py8qg3e/v1. In summary, high molecular weight (HMW) DNA was isolated from 1mL of frozen whole blood using the KingFisher Apex instrument, following an adapted version of the PacBio Nanobind HT HMW DNA Extraction 1mL Whole Blood KingFisher Apex protocol, along with the Nanobind HT 1mL Blood Kit (102-762-800) from PacBio. The DNA underwent a size selection step with the PacBio Short Read Eliminator Kit (102-208-300) to eliminate fragments up to 25kb. Subsequently, the DNA was sheared to a target size of 30kb using the Megaruptor 3 instrument and the Diagenode Megaruptor 3 Shearing Kit (E07010003). The DNA library was then prepared using the SQK-LSK114 Ligation Sequencing Kit from Oxford Nanopore Technologies (ONT). The samples were loaded onto a PromethION R10 flow cell (FLO-PRO114M) according to ONT’s standard operating procedures and run for 72 hours on a PromethION device.

Fast5 files containing raw signal data were acquired from sequencing conducted with Oxford Nanopore Technologies’ MinKNOW v22.10.7 (https://nanoporetech.com/about-us/news/introducing-new-minknow-app). Super accuracy base calling was performed on all Fast5 files for each sample using Oxford Nanopore Technologies’ Dorado v0.6.0 (RRID:SCR_025883). Fastq files that passed quality control filters during the super accuracy basecalling step were subsequently aligned to the hg38 reference genome using Winnowmap (RRID:SCR_025349). The resulting SAM files were sorted, converted to BAM files, and indexed with SAMtools v1.20 (RRID:SCR_002105)^34^. These BAM files were then merged, sorted, and indexed to generate a final BAM file for each sample. Structural variants (SVs) were detected and genotyped using Sniffles2 v.2.3 (RRID:SCR_017619, https://github.com/fritzsedlazeck/Sniffles)^35^.

#### Pipeline

##### Model Framework

Our pipeline employs a recurrent neural network known as Long Short-term Memory (LSTM)^36^, known for its proficiency in capturing long-term dependencies within sequential data. We stack three LSTM layers which feed sequence outputs to subsequent layers, creating a deeper network that learns multiple levels of abstraction for improved feature extraction. A hard sigmoid activation function was chosen for its decreased computational cost and success with deep learning binary classification tasks. Therefore, our model outputs predicted values between 0 and 1 which correspond to the likelihood of a CNV for that sample in the region of interest (Supplementary Figure 1).

Due to our model architecture, we apply overlapping sliding windows to the signal intensity files during data preparation for our model. To determine window sizes for each gene or interval of interest, we utilize customizable variables for split and total window counts within the region. For instance, if a model is trained with a split of 5 and a total window count of 50, this signifies that a gene like *PRKN* is divided into 5 equal segments, each spanning 276,049 base pairs without any overlap. Subsequently, 50 windows are computed to overlap these segments by 22,534 bases. Similarly, in *MAPT*, the 5 equal intervals would be 26,790 base pairs long with windows overlapping by 2,168 bases. This approach creates proportional windows to span any gene regardless of its size, without the need for padding with artificial bases to create sequences of equal lengths. Furthermore, it enables the application of a single model to all regions prepared with the same split and window counts. Additionally, we include a modifiable buffer that can be added to both sides of a region of interest before window calculation. In our analysis, we incorporated a default 250 kb buffer to facilitate the exploration of variant behavior surrounding the regions of interest, which we later customized for the analyses of *APP* and *SNCA*.

**Figure 1.**
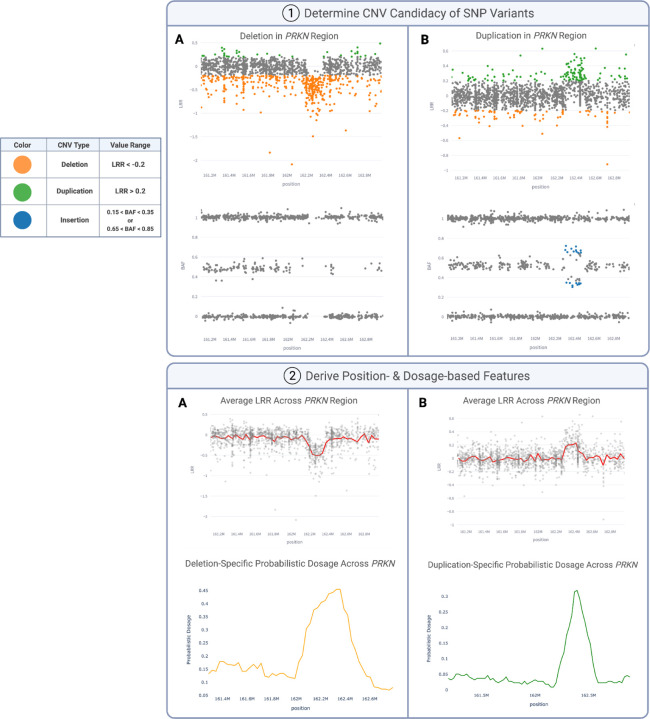
Feature creation to capture deletions (A) and duplications (B). (1) Illumina-based thresholds are applied to SNPs to determine if their LRR and BAF values fall into the proper ranges to be considered deletions and duplications. (2) Features in Supplementary Table 6 are calculated on the variants within these specified CNV ranges (CNV candidates).

Features summarized in Supplementary Table 6 are aggregated within and across each window to capture trends such as visible positional shifts in LRR or BAF and probabilistic dosage spikes. These spikes are depicted in Figure 1 by LRR or BAF against chromosome position (in base pairs) for *PRKN* deletions (Column A) and duplications (Column B). SNP variants in this figure, color-coded for visibility, are deemed “CNV candidates” if they fall within specific ranges recommended by the manufacturer:

- Deletions: LRR < −0.2
- Duplications: LRR > 0.2
- Insertions: 0.15 < BAF < 0.35 or 0.65 < BAF < 0.85

To comprehensively capture the mid-range of BAFs, we incorporate additional features encompassing BAF values from 0.15 to 0.85, inclusive. This broader range of frequencies is crucial for capturing characteristics such as the diverging branching pattern consistently observed in duplications of varying size and BAF values^37^. Most model features are only derived from the CNV candidates, aiming to enhance precision and reduce computational cost by subsetting the extensive pool of SNPs within each region.

A five-fold cross-validation was performed on the training set to ascertain optimal window sizes and feature combinations for predicting each CNV type. A split of 5, sliding window of 50, and the 4th feature combination, earned an average Area Under the Curve (AUC) (Supplementary Table 7) of 0.93 for our deletion model. For the duplication model, an average AUC of 0.92 was obtained with a split of 10, sliding window of 70, and the 6th feature combination. Therefore, if you choose to utilize our existing models for a cohort of size N samples, the pipeline will reshape the model-ready input files to the necessary dimensions of [N, 50, 13] and [N, 70, 11] for the deletion and duplication models, respectively.

##### Semi-Automated Integration with Visualization

The final step of CNV-Finder prepares app-compatible files for visualization within our interactive web application. This interface enables researchers to review predictions and filter displayed samples by model prediction values, LRR range, and variant count in order to explore or confirm results. Users have the flexibility to examine results by selecting from options such as “Yes,” “Maybe,” or “No,” which are used to record chosen sample IDs, their respective regions of interest, and the type of CNV under evaluation. A sample can also be marked accordingly if a different class of structural variants is observed in the plots. Logs generated by the application can subsequently serve as input to the pipeline for enlarging the training set and training a new model. To prevent data leakage if our model is applied to the GP2 cohorts that were used in our training sets, all overlapping samples are automatically excluded from testing sets in repeated regions.

## Results

CNV-Finder facilitated the preparation of genotyping data, visual assessment of predicted CNV-carriers, and re-training of two additional models per CNV type improving on the preliminary models trained on 184 expert-annotated samples (refer to Supplementary Table 2). Each set of three models per CNV type shared identical architectures and varied only in training set size, with the final deletion and duplications models incorporating the most samples. Upon application of the preliminary model to five cohorts (BBDP, CAT-PD, LCC, MDGAP-KINGS, SYDBB in Table 1) and evaluation of candidates with predicted values equal to or exceeding 0.8, 116 samples (31 deletions, 85 negative samples) were incorporated into our deletion training set, while 354 samples (132 duplications, 222 negative samples) were added to our duplication training set. At this stage, the updated training sets consisted of 82% *PRKN* deletions, 18% *LINGO2* deletions, 79% *MAPT* duplications, and 21% *PRKN* duplications. Subsequently, the updated model was applied to the held-out, large-scale CORIELL cohort augmenting the final training sets with 159 samples (150 deletions, 9 negative samples) and 126 samples (53 duplications, 73 negative samples) for deletions and duplications, respectively. Our final training sets comprised 71% *PRKN* deletions, 29% *LINGO2* deletions, 59% *MAPT* duplications, and 41% *PRKN* duplications. Evaluation of the final model was conducted on PPMI, with validation from short and long-read whole genome sequencing. The final models were then applied to the non-locally-restricted samples from GP2’s 7th data release (https://gp2.org/the-components-of-gp2s-seventh-data-release/) on all genes of interest, including *SNCA* and *APP*, to achieve the final phenotypic counts per region.

Development of the preliminary, updated, and final models involved over 12,500 samples while the application on GP2’s 7th data release included roughly 44,000 samples. Benchmarking was conducted on 720 samples within the BBDP cohort in the *PRKN* gene locus. All cohorts and genes can be processed in parallel, potentially benefiting from enhanced processing efficiency with additional CPUs. For instance, data processing and feature aggregation, the most computationally intensive tasks, yielded a model-ready 16 MB file for 70 overlapping windows in approximately 23 minutes with 1 CPU and 7.5 minutes with 8 CPUs. With 1 CPU, model testing on BBDP samples took only 19 seconds and model training on the final and largest training set of 594 samples generated a 1 MB model file after running for 31 seconds.

### Summary of CNV Disease Associated Regions

Among the GP2 samples included in our analysis, 710 deletions were found in *PRKN, LINGO2*, and *APP* (Table 2). Although *APP* duplications are of particular interest, only strong deletion candidates were detected in our dataset due to the rarity of these variants^11^. Otherwise, 1,913 duplications were identified in *PRKN*, *MAPT*, and *SNCA* (Table 3). Each of these SVs were visually confirmed using the CNV-Finder app. The phenotype counts indicate that most samples with detected CNVs were diagnosed with PD across all genes of interest, except for *LINGO2*, where the majority of samples with deletions were controls. We did not distinguish between monogenic or sporadic forms of PD in our datasets. The control group for all regions also includes a small subset of unaffected Leucine-rich repeat kinase 2 (*LRRK2*) positive samples and individuals with microscopic Alzheimer’s disease lesions that were insufficient for diagnosis. The “Other” phenotypic category encompasses disorders such as Parkinsonism or Scans Without Evidence of Dopaminergic Deficit (SWEDD) Syndrome. The “Mix” category includes any combination of NDD diagnoses, with the “LBD” category more specifically representing a combination of PD and DLB. As we scale up our analyses, we can more effectively determine the clinical significance of SVs within specific regions of interest.

## Discussion

### Visual Validation

Even with its smallest training set, CNV-Finder correctly identified, with predicted values above 0.5, 97% of the held-out CORIELL deletions and 84% of the duplications within *PRKN* (Supplementary Table 8). Subsequent sample additions to the training set reduced false positives of the deletion model and decreased false negatives of the duplication model, while maintaining high rates of true positives in the updated model. Consequently, this improvement led to increases in the AUC metrics, from 0.92 to 0.99 for deletions and 0.91 to 0.92 for duplications, as shown in Supplementary Table 8.

Furthermore, the updated models effectively reduced the overall number of predicted CNV samples within the full CORIELL cohort. This therefore reduced the workload for human confirmation, during the sample review process in our application, to a significantly smaller subset of predicted candidates. Initially, the preliminary deletion and duplication models predicted 925 and 333 samples with scores over 0.8, respectively. The updated deletion and duplication models reduced this to only 100 and 126 samples with predictions over 0.8. Among the 100 *PRKN* samples with predicted values exceeding 0.8 for deletions, 96 samples exhibited visible CNVs and were consequently integrated into the final deletion training set. Similarly, from the pool of 126 *PRKN* duplication candidates with prediction values of at least 0.8, the final duplication training set incorporated 53 samples, all of which attained prediction values exceeding 0.9. Supplementary Figure 2 showcases diverse, non-standard examples detected within CORIELL with predicted values of 1 to demonstrate the generalizability of our models. These samples include homozygous deletions and noisy duplications which later enrich the feature spectrum captured by our training sets. Our goal with the updated training sets was to increase the overall accuracy and further reduce the Brier score (Supplementary Table 7) for both models.

### Short- and Long-Read Sequencing Validation

**Figure 2.**
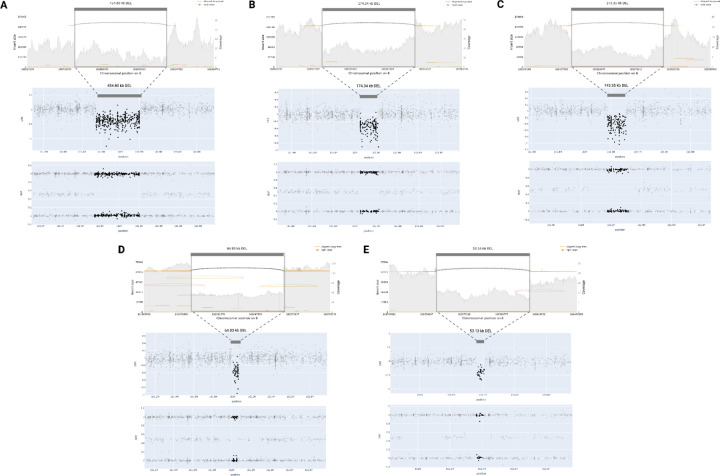
Long-read WGS validation of predicted deletions in *PRKN*. Four samples (A, B, C, E) received prediction values of 1 and one sample (D) received 0.93.

**Figure 3.**
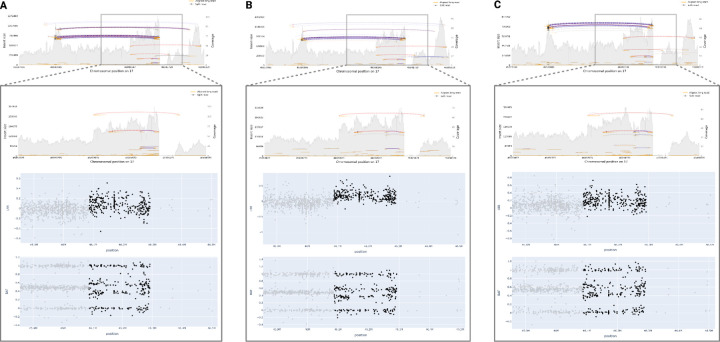
Long-read WGS validation of predicted duplications near *MAPT*. All three samples received prediction values of 1.

We used both Illumina short-read and ONT long-read WGS to validate the predicted samples. Long-read data, in particular, can identify CNVs that are distinct from what short reads and arrays can detect^2^. Short-read data is constrained by relatively high rates of false positives, making it challenging to calculate a false negative rate and potentially skewing true positive rates with respect to CNV-Finder’s results^38^. Our focus was on validating samples with prediction values exceeding 0.9 using ONT long-read WGS data to ensure that the strongest candidates, representing the most objective examples from our models, were not false positives. Figure 2 showcases five samples with predicted and confirmed *PRKN* deletions by long-read sequencing. These deletions vary in size, ranging from approximately 53 kb to 455 kb. Additionally, Figure 3 features three duplications near *MAPT* validated by long-read sequencing. As previously mentioned, a 250 kb buffer surrounding the gene interval was employed in our analyses, enabling us to capture these duplications in the regions overlapping *MAPT* and *KANSL1*. Despite variations in the exact shape and variant noise levels, all validated CNVs received predicted values of 1.0 from their respective models, with only one predicted deletion obtaining a value of 0.93.

**Figure 4.**
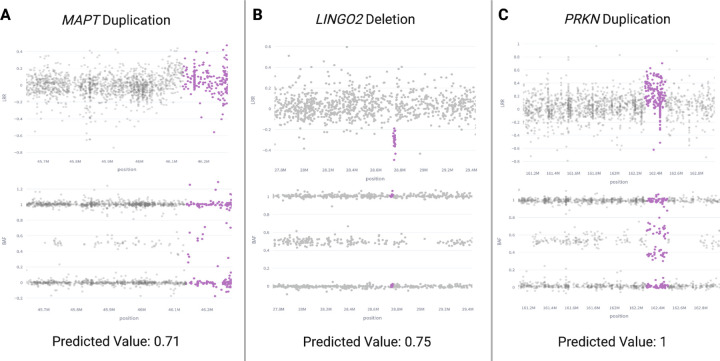
Short-read sequencing validation of model results with varied prediction values.

To complement the limitations of having long-read WGS data for only 26 samples, we utilized short-read sequencing of 337 samples to illustrate CNVs with varying model prediction values in Figure 4. The highlighted pink variants denote areas of deletions or duplications called in the short-read data. Model prediction values and visual clarity of the confirmed CNVs increase from left to right in the figure. Notably, *LINGO2* features the smallest deletion captured and confirmed by the model, with a width of only about 27 kb.

Supplementary Tables 9 and 10 summarize all confirmed samples for each of the four use cases involved in model creation with long-read and short-read validation, respectively. False negatives are inflated due to the nature and granularity of the varying genetic modalities.

Brier scores computed only with respect to the true positive, true negative, and false positive samples, demonstrate low loss values. Brier score is a measure of accuracy for probabilistic predictions, where lower values denote lower loss, indicating better performance (Supplementary Table 7). Therefore, the inclusion of false negatives in the calculation led to a significantly increased Brier score which remained consistently high despite successive model improvements. This underscores the limitations of array-based data in detecting structural variants identified by more advanced sequencing technology, while highlighting strong performance on positive predictions.

Positive Predictive Value (PPV) and Negative Predictive Value (NPV) metrics (Supplementary Table 7) also reflect this trend. For short-read comparisons, PPV values averaged 0.95 among the final two deletion model results and 0.9 for the final two duplication model results. The average NPV is notably lower for deletions at 0.07 and 0.8 for duplications. PPV scores remained high with long-read validation, demonstrating average scores of 1 for *PRKN* deletions (*LINGO2* deletions were excluded due to insufficient samples) and 1 for *MAPT* duplications, while averaging 0 for deletions and 0.17 for *MAPT* duplications (*PRKN* duplications were excluded due to insufficient samples).

### Duplications within the 17q21.31 Region

Further exploration of the complex *MAPT* region highlights the crucial role of contextual understanding and human proficiency in enhancing the precision of CNV identification. Deliberate consideration was given to the classifications of “Yes,” “Maybe,” and “No” within the CNV-Finder application when selecting samples to add to the training set. Supplementary Figure 3 showcases examples corresponding to each selection category. Samples D and E in this figure were marked as “Maybe” to avoid mislabeling them as negative samples with no CNVs. Typical duplication patterns are discernible in their LRR vs. position plots while the BAF vs. position plots lacked sufficient data. Additionally, potential artifacts like Sample F were marked as “Maybe” to prevent inclusion in the training sets. The duplication pattern highlighted in the “Yes” column, consistently appearing at locations around 461 kb to 463 kb on the 17th chromosome near *MAPT* and within the *KANSL1* region, exhibited the highest prevalence among the inspected CNVs across our three genes of interest. This duplication manifested in diverse phenotypes across our use cases, as summarized in Tables 2 and 3. Across all testing cohorts, a total of 737 samples exhibited visible duplications, while 231 samples showed no or limited evidence of duplication in the *MAPT* region, resulting in the annotation of 1151 samples with “Yes” or “No” for this variant.

As previously mentioned, the 17q21.31 region which encompasses the *MAPT* gene, is made complex by an H1 haplotype and inverted H2 haplotype. To better analyze this region and distinguish between haplotypes, we examined the H1/H2 tagging SNP rs8070723 across the 1151 annotated samples, revealing a Jaccard similarity score (Supplementary Table 7) of 0.94 between H2-carrier status (H1H2+H2H2) and the presence of an obvious duplication in the region (“Yes” classification). A logistic regression analysis was conducted on an 80:20 partition of these samples, ensuring consistency in the distributions of population ancestries across both data subsets. This resulted in a 97% accuracy and an AUC of 0.95 when predicting the existence of a duplication in this region using only H2-carrier status as the feature. There were only 6 false positives and 0 false negatives in the test set comprising 146 samples with the observed duplication and 85 samples without.

Furthermore, to better examine this variant in H2 non-carriers (H1H1), we applied our final duplication model to AD and dementia cases in the MDGAP-DEMENTIA and REasons for Geographic and Racial Differences in Stroke (REGARDS) cohorts (Supplementary Table 5). Among the 506 cases included in these cohorts, H2 non-carriers constitute 64%. Upon visual inspection of all samples with predicted values over 0.5, 39 instances were found to meet the criteria typically associated with a “Yes” designation in Supplementary Figure 3. These instances encompass a variety of NDDs, including cases of AD, FTD, PSP, DLB, MSA, CBD/CBS, Undetermined-Dementia, and other tauopathies; however, all are H2-carriers. Conversely, out of the 84 samples annotated as “Maybe” due to sparse BAF vs. chromosome position plots resembling those seen in plots D and E of Supplementary Figure 3, all are H2 non-carriers (H1H1). One sample reported as a “No Call” for SNP rs8070723 was categorized as “No.” Had the duplication model not been trained with careful consideration for the expansion of both binary classes (CNV exists vs. CNV does not exist), the final model would not have assigned high probabilities to H2 non-carriers, thus neglecting their flagging for human review. This highlights the significance of contextual interpretation by researchers and the additional layer of insight their expertise provides, particularly when array-based plots may not fully reflect all pertinent information.

### Utility

CNV-Finder facilitates streamlined and comprehensive analysis of deletions and duplications, capable of handling sample sizes necessary for capturing rare SVs and enhancing the statistical power of disease associations. This pipeline operates independently of outputs from any supplementary CNV prediction or imaging software, harnessing the extensive advantages of contemporary deep learning models. Additionally, its optional semi-automated functionality allows for the integration of expert validation, a process traditionally essential for confirming predicted candidates from standard array-based callers. Human expertise proves invaluable, particularly in complex regions like 17q21.31, where additional context is needed for accurate interpretation of detected variants or for navigating artifacts that may mimic structural variant features. Models trained with user visual confirmation have demonstrated enhanced detection capabilities for noisy and small CNVs, concurrently reducing false positive rates.

### Limitations and Next Steps

It is noteworthy that while human validation within our semi-automated approach has enhanced model performance in our analyses, it can also introduce inconsistencies and biases to predictions. The balance and representation of each class (samples with CNVs vs. samples without CNVs) in the training set may be influenced by selections in a manner detrimental to prediction accuracy. Furthermore, as the models advance in CNV detection, adding negative samples to the training set with prediction values exceeding 0.8 may become increasingly challenging.

In addition, our use cases have been confined to regional explorations with binary outputs from a fixed-length, sequence-to-vector LSTM model. This decision was made to facilitate rapid predictions on gene regions with established significance or interest demonstrated in the literature. Future iterations of the model will leverage LSTMs’ capacity to generate sequences of model prediction values. Although this adjustment may increase computational cost, it could enable users to apply the model across the entire genome, with outputs indicating more specific regions of elevated probability for each CNV type. We may also introduce new features that consider region-specific SNPs, such as the *MAPT* H1/H2 tagging variant, to provide additional context to our predictions.

Future studies will need to investigate the performance across a wider spectrum of genotyping arrays, which may exhibit varying levels of call quality and coverage of genetic variants. Moreover, when compared to more detailed modalities, such as long-read sequencing, we may artificially deflate the false positive rate of our model due to the detection of small variants that are missed by array-based approaches. It is imperative to incorporate human validation into these comparisons to ascertain whether the model could have even discerned visible patterns in the data. Beyond this, the nature of our model architecture and its use of sliding windows, make it possible to transition our models to work on short-read WGS and potentially improve predictions through the enhancements in call quality from this modality.

Finally, as previously reported, the 17q21.31 region challenges the accuracy of short- and long-read SV calling^33^. Conventional variant calling algorithms often struggle to precisely determine the position ranges of potential CNVs within this genomic region. For instance, Sniffles2, a widely-used open-source tool for analyzing long-read SVs, identified duplications in our PPMI samples proximal to *MAPT*, spanning positions 46135410 to 46292247 on chromosome 17, across both short-read and long-read sequencing data. However, upon visual examination of the three assessed samples (referenced in Figure 3) alongside SAMtools plots derived from long-read sequencing data, it became apparent that the start of the CNV likely occurred earlier than reported. Moreover, the loss of sequencing coverage immediately following the duplication complicates the accurate determination of the breakpoint. Regardless, our results align with prior research in this genomic locus, affirming the importance of investigating the genetic significance and phenotypic consequences of these identified duplication candidates^39^.

## Conclusion

This study presents a comprehensive pipeline integrating deep learning techniques to expedite large-scale identification of CNVs within predefined genomic regions. Through application across six distinct use cases involving SVs within five genes implicated in NDD susceptibility, we showcase the adaptability and robustness of both our deletion and duplication models. By merging model predictions with domain-specific expertise, our approach significantly reduces the burden of manual validation, facilitating the identification of precise CNV candidates for subsequent downstream analyses or targeted validation using resource-intensive methods such as PCR or long-read sequencing.

## Supplementary Material

Supplement 1

Supplement 2

## Figures and Tables

**Table 1.** Summary statistics of non-locally-restricted GP2 cohorts used in testing and re-training. These groups include the Arizona Brain Bank Brain and Body Donation Programme (BBDP), Central Asian and Transcaucasian PD (CAT-PD), LRRK2 Cohort Consortium (LCC), Defining and diagnosing neurodegenerative Movement Disorders through integrated analysis of Genetics and neuroPathology at King’s College (MDGAP-KINGS), and the Sydney Brain Bank (SYDBB) groups. Bolded cohorts with asterisks denote the availability of extension data to validate model prediction, including expert-identified CNVs in CORIELL (Supplementary Table 3) and short- and long-read WGS data for PPMI samples (Supplementary Table 4). “Undetermined-MCI” represents undiagnosed mild cognitive impairment and “Prodromal” represents prodromal PD with no motor symptoms. “MSA” refers to Multiple System Atrophy.

*Cohort*	Diagnosis	Median Age	Males	Females	Unknown Sex	Total
*BBDP*	Mix	76	153	95		248
	Control	85	120	106		226
	PD	78	111	43		154
	Undetermined-MCI	90	38	33		71
	DLB	80	14	5		19
	AD	72	0	1		1
*CAT-PD*	PD	62	149	190		339
	Control	53	172	150		322
	Other	39	2	1		3
	PSP	74	1	0		1
*LCC*	Control	52	86	129		215
	PD	67	79	69		148
*MDGAP-KINGS*	PD	78	65	34	4	103
	DLB	82	24	11		35
*SYDBB*	PD	63	60	28		88
	DLB	72	23	7		30
***CORIELL*** *	PD	63	2784	1657	2	4443
	Control	67	1779	2249		4028
	PSP	65	195	178		373
	MSA	61	27	26		53
	DLB	69	24	11		35
	Other	55	10	13		23
***PPMI*** *	PD	67	443	295		738
	Control	62	337	395		732
	Prodromal	68	53	43		96
	Other	62	34	22		56
						**12580**

**Table 2.** Phenotype counts of all non-locally-restricted samples from GP2’s 7th data release with visually-confirmed deletions in genes of interest. Proportions of the total population are included in parentheses per diagnosis. “VaPD” stands for Vascular Parkinsonism.

Visually-Confirmed Deletions				
Diagnosis	Total Population	*PRKN*	*LINGO2*	*APP*
PD	23,089	236 (1.0%)	162 (0.7%)	7 (0.03%)
Control	13,366	91 (0.7%)	119 (0.9%)	5 (0.03%)
Population Control	5,458	25 (0.5%)	35 (0.6%)	0
PSP	760	7 (0.9%)	2 (0.3%)	0
Other	382	2 (0.5%)	2 (0.5%)	0
DLB	296	1 (0.3%)	2 (0.7%)	0
Mix	252	1 (0.4%)	6 (2.4%)	0
MSA	243	4 (1.6%)	0	0
Prodromal	120	0	1 (0.8%)	0
AD	114	1 (0.9%)	0	0
LBD	75	0	0	0
Undetermined-MCI	71	1 (1.4%)	0	0
Undetermined-Dementia	27	0	0	0
CBD/CBS	23	0	0	0
FTD	3	0	0	0
VaPD	1	0	0	0
**Total**	**44,280**	**369**	**329**	**12**

**Table 3.** Phenotype counts of all non-locally-restricted samples from GP2’s 7th data release with visually-confirmed duplications in genes of interest. Proportions of the total population are included in parentheses per diagnosis.

Visually-Confirmed Duplications				
Diagnosis	Total Population	*PRKN*	*MAPT*	*SNCA*
PD	23,089	82 (0.4%)	890 (3.9%)	24 (0.1%)
Control	13,366	42 (0.3%)	401 (3.0%)	2 (0.01%)
Population Control	5,458	16 (0.3%)	336 (6.2%)	0
PSP	760	0	6 (0.8%)	0
Other	382	3 (0.8%)	22 (5.8%)	0
DLB	296	0	15 (5.1%)	0
Mix	252	0	13 (5.2%)	0
MSA	243	1 (0.4%)	26 (10.7%)	0
Prodromal	120	1 (0.8%)	3 (2.5%)	0
AD	114	0	10 (8.8%)	0
LBD	75	0	5 (6.7%)	0
Undetermined-MCI	71	0	8 (11.3%)	0
Undetermined-Dementia	27	0	3 (11.1%)	0
CBD/CBS	23	1 (4.3%)	2 (8.7%)	0
FTD	3	0	1 (33.3%)	0
VaPD	1	0	0	0
**Total**	**44,280**	**146**	**1,741**	**26**

## Data Availability

This pipeline is implemented in Python and applied in Jupyter notebooks available for reference at: https://github.com/GP2code/CNV-Finder; DOI 10.5281/zenodo.14182563. Further documentation is included in this repository with all pre-trained models saved in the CNV-Finder/ref_files/models folder.
