# Supplementary material for: CNV-Finder: Streamlining Copy Number Variation Discovery
Source: bioRxiv. 2025 Jul 26:2024.11.22.624040. Originally published 2024 Nov 23. Preprint. [Version 2] doi: 10.1101/2024.11.22.624040 (PMC11601614; doi:10.1101/2024.11.22.624040)
Supplement: Supplement 1 — Supplementary Table 1. Descriptions of GP2 cohorts involved in training and validating our models. Supplementary Table 2. Summary of training set from expert annotations of PRKN interval. (A) PRKN deletions (B) PRKN duplications (C) Negative samples with no CNVs. Supplementary Table 3. Summary statistics of GP2 cohorts used in training our updated and final models. “Undetermined-MCI” represents undiagnosed mild cognitive impairment and “Prodromal” represents prodromal PD with no motor symptoms. “MSA” refers to Multiple System Atrophy. Supplementary Table 4. Summary statistics of held-out expert annotations for validation of preliminary and updated models on CORIELL samples. CORIELL samples were incorporated in the training set of our final models. Supplementary Table 5. Summary statistics of PFP and STELLENBOS samples with MLPA data for prediction validation. Supplementary Table 6. Summary of PPMI extension data available for prediction validation. No samples described here overlap with any PPMI samples incorporated in the models’ training sets. Supplementary Table 7. Summary statistics of additional NDD phenotype cases. Supplementary Table 8. Descriptions of features and tested combinations used in model training. “Avg.” stands for average, “IQR” represents interquartile range, and “SD” refers to standard deviation. Supplementary Table 9. Descriptions of statistical metrics used in assessing model performance. Supplementary Table 10. Preliminary and updated model results for known PRKN CNVs in held-out CORIELL PD Cases. Visually-confirmed samples with predicted values above 0.8 were fed back into the updated model’s training set to create the training sets for our final models. Supplementary Table 11. Summary of model predictions on combined MLPA data from PFP and STELLENBOS cohorts. Asterisks denotes that the final duplication model was used to make predictions on SNCA; however, both duplications and triplications were identified. Supplementary Table 12. Summary o [file media-1.pdf]

Cohort	Description
APGS	Australian Parkinsons' Genetics Study
BBDP	Arizona Brain Bank Brain and Body Donation Programme
BCM	Baylor College of Medicine
CAT-PD	Central Asian and Transcaucasian Parkinson's Disease
COPN	Canadian Open Parkinson's Network
CORIELL	Coriell Institute for Medical Research
IPDGCAF-NG	International Parkinson Disease Genomics Consortium Africa
KOC	Koc University
LCC	LRRK2 Cohort Consortium
MDGAP-DEMENTIA	Defining and diagnosing neurodegenerative Movement Disorders through integrated analysis of Genetics and neuroPathology [Not part of GP2 data releases]
MDGAP-KINGS	Defining and diagnosing neurodegenerative Movement Disorders through integrated analysis of Genetics and neuroPathology at King's College
MDGAP-QSBB	Defining and diagnosing neurodegenerative Movement Disorders through integrated analysis of Genetics and neuroPathology at University College London
NZP3	New Zealand Parkinson's Programme
PAGE	Parkinson's Genes and Environment Study
PDGNRTN	PDGENEration at the Parkinson's Foundation in Miami
PFP	Parkinson's Families Project
PPMI	Parkinson's Progression Markers Initiative
REGARDS	REasons for Geographic and Racial Differences in Stroke
STELLENBOS	Parkinson's Disease in Southern Africa
SYDBB	Sydney Brain Bank
UMD	University of Maryland
UMKLM	Malaysian Parkinson's Genetics Cohort

Supplementary Table 1. Descriptions of GP2 cohorts involved in training and validating our models.

A

Positive Class - PRKN Deletions

Cohort	**Diagnosis**	**Median Age**	**Males**	**Females**	**Total**
APGS	PD	-	7	7	14
STELLENBOS	PD	55	8	3	11
BCM	PD	-	7	2	9
PAGE	PD	76	6	3	9
COPN	PD	67	6	0	6
PDGNRTN	PD	55	2	3	5
MDGAP-QSBB	PD	72	4	0	4
NZP3	PD	45	2	2	4
IPDGCAF-NG	PD	-	3	0	3
UMD	PD	-	0	2	2
BBDP	PD	77	1	0	1
KOC	PD	52	0	1	1
UMKLM	PD	54	1	0	1
					**70**

B

Positive Class - PRKN Duplications

Cohort	**Diagnosis**	**Median Age**	**Males**	**Females**	**Total**
PDGNRTN	PD	67	2	5	7
PAGE	PD	72	4	2	6
APGS	PD	-	3	2	5
COPN	PD	66	2	2	4
STELLENBOS	PD	60	1	2	3
UMD	PD	-	2	1	3
IPDGCAF-NG	PD	-	1	0	1
					**29**

C

Negative Class - No CNVs in PRKN

Cohort	**Diagnosis**	**Median Age**	**Males**	**Females**	**Total**
PPMI	PD	62	34	23	57
	Control	64	20	8	28
					**85**

Supplementary Table 2. Summary of training set from expert annotations of *PRKN* interval. (A) *PRKN* deletions (B) *PRKN* duplications (C) Negative samples with no CNVs.

Cohort	Diagnosis	Median Age	Males	Females	Unknown Sex	Total
BBDP	Mix	76	153	95		248
	Control	85	120	106		226
	PD	78	111	43		154
	Undetermined-MCI	90	38	33		71
	DLB	80	14	5		19
	AD	72	0	1		1
CAT-PD	PD	62	149	190		339
	Control	53	172	150		322
	Other	39	2	1		3
	PSP	74	1	0		1
CORIELL	PD	63	2784	1657	2	4443
	Control	67	1779	2249		4028
	PSP	65	195	178		373
	MSA	61	27	26		53
	DLB	69	24	11		35
	Other	55	10	13		23
LCC	Control	52	86	129		215
	PD	67	79	69		148
MDGAP-KINGS	PD	78	65	34	4	103
	DLB	82	24	11		35
SYDBB	PD	63	60	28		88
	DLB	72	23	7		30
						**10,958**

Supplementary Table 3. Summary statistics of GP2 cohorts used in training our updated and final models. "Undetermined-MCI" represents undiagnosed mild cognitive impairment and "Prodromal" represents prodromal PD with no motor symptoms. "MSA" refers to Multiple System Atrophy.

A

***PRKN* Deletions**

Cohort	Diagnosis	Median Age	Males	Females	Total
CORIELL	PD	55	99	51	150

B

***PRKN* Duplications**

Cohort	Diagnosis	Median Age	Males	Females	Total
CORIELL	PD	59	28	10	38

C

Negative Class - No CNVs in *PRKN*

Cohort	Diagnosis	Median Age	Males	Females	Total
CORIELL	PD	64	2621	1523	4,144

Supplementary Table 4. Summary statistics of held-out expert annotations for validation of preliminary and updated models on CORIELL samples. CORIELL samples were incorporated in the training set of our final models.

Cohort	Diagnosis	Median Age	Males	Females	Total
PFP	PD	62	422	322	744
	Control	64	5	10	15
	Other	59	2	0	2
STELLENBOS	PD	67	274	196	470
	Control	24	2	1	3
					**1,234**

Supplementary Table 5. Summary statistics of PFP and STELLENBOS samples with MLPA data for prediction validation.

Data Type	Diagnosis	Median Age	Males	Females	Total
Short-read WGS	PD	65	154	73	227
	Control	64	71	37	108
	Other	66	2	0	2
					**337**
Long-read WGS	PD	68	11	6	17
	Control	66	2	5	7
	Prodromal	67	0	1	1
	Other	52	1	0	1
					**26**

Supplementary Table 6. Summary of PPMI extension data available for prediction validation. No samples described here overlap with any PPMI samples incorporated in the models' training sets.

Cohort	Diagnosis	Median Age	Males	Females	Unknown Sex	Total
MDGAP-DEMENTIA	AD	59	52	55	3	110
	PSP	71	62	32		94
	FTD	58	42	25	1	68
	Other	67	21	14	7	42
	DLB	61	28	13		41
	MSA	65	8	7		15
	CBD/CBS	67	4	4		8
	PD	51	0	1		1
REGARDS	AD	75	60	40		100
	Undetermined-Dementia	75	14	13		27
						**506**

Supplementary Table 7. Summary statistics of additional NDD phenotype cases.

Feature Name Calculated per Window	Description
Dosage Full	Ratio of CNV candidates in window to total number of CNV candidates across full region of interest
CNV-specific Dosage Full	Ratio of specific CNV candidates (either deletions or duplications) in window to total number of CNV candidates across full region of interest
CNV-specific Dosage Interval	Ratio of specific CNV candidates (either deletion or duplication) in window to total number of all variants in that window
BAF Std. Dev.	Standard deviation of B Allele Frequencies within window
Mid-BAF Std. Dev.	Standard deviation of B Allele Frequencies (>= 0.15 & <=0.85) within window
LRR Std. Dev.	Standard deviation of Log R Ratio within window
BAF IQR	Interquartile range of B Allele Frequencies within window
Mid-BAF IQR	Interquartile range of B Allele Frequencies (>= 0.15 & <=0.85) within window
LRR IQR	Interquartile range of B Allele Frequencies within window
Avg. BAF	Average B Allele Frequencies within window
Avg. Mid-BAF	Average B Allele Frequencies (>= 0.15 & <=0.85) within window
Avg. LRR	Average Log R Ratio within window
**Feature Combination**	**Features Included**
Combination 1	Dosage Interval, CNV-specific Dosage Full, Avg. BAF, Avg. LRR
Combination 2	Dosage Interval, Dosage Full, CNV-specific Dosage, BAF SD, LRR SD, BAF IQR, LRR IQR, Avg. BAF, Avg. LRR
Combination 3	Dosage Interval, Dosage Full, CNV-specific Dosage Full, CNV-specific Dosage Interval, BAF SD, LRR SD, BAF IQR, LRR IQR, Avg. BAF, Avg. Mid BAF, Avg. LRR
Combination 4	Dosage Interval, Dosage Full, CNV-specific Dosage Full, CNV-specific Dosage Interval, BAF SD, Mid BAF SD, LRR SD, BAF IQR, Mid BAF IQR, LRR IQR, Avg. BAF, Avg. Mid BAF, Avg. LRR
Combination 5	Dosage Interval, Dosage Full, CNV-specific Dosage Full, BAF SD, Mid BAF SD, LRR SD, BAF IQR, Mid BAF IQR, LRR IQR, Avg. BAF, Avg. Mid BAF, Avg. LRR
Combination 6	Dosage Interval, CNV-specific Dosage Full, BAF SD, Mid BAF SD, LRR SD, BAF IQR, Mid BAF IQR, LRR IQR, Avg. BAF, Avg. Mid BAF, Avg. LRR

Supplementary Table 8. Descriptions of features and tested combinations used in model training. “Avg.” stands for average, “IQR” represents interquartile range, and “SD” refers to standard deviation.

**Statistical Metric**	**Description**
Area Under the Curve (AUC)	Demonstrates performance of a classification model by representing how well the model can separate and distinguish the classes. In our case, we are evaluating how accurately our model outputs a predicted value above 0.5 when a CNV exists in the region of interest for each sample.
Brier Score	Evaluates the accuracy of predicted probabilities of an event with a range of 0 to 1, with a score of 0 representing perfect accuracy. This score reveals the accuracy of our models' predicted values of a CNV existing when compared to the actual existence of the SV. It allows us to see if the predicted value output by the model correlates with the strength of CNV candidates, meaning that the most easily observable deletions and duplications should receive predicted probabilities above 0.9.
Positive Predictive Value (PPV)	Represents the likelihood that a sample with a predicted value of 0.5 from our models, would have a CNV present in the region of interest.
Jaccard Similarity Score	Measures the similarity between two datasets by dividing the intersection of both sets by their union. A score of 1.0 implies that two datasets include the same members; therefore in our "Duplications within the 17q21.31 Region" analysis, the score of 0.94 implies that the H2-carrier status was "Yes" in almost every instance that the presence of an obvious duplication in the 17q21.31 region was "Yes."

Supplementary Table 9. Descriptions of statistical metrics used in assessing model performance.

Deletions

	Preliminary Model	Updated Model
True Positives (TP)	146	147
True Negatives (TN)	3607	4103
False Positives (FP)	537	41
False Negatives (FN)	4	3
Brier Score	0.114	0.055
Sensitivity	0.97	0.98
Specificity	0.87	0.99
AUC	**0.92**	**0.99**

Duplications

	Preliminary Model	Updated Model
True Positives (TP)	32	33
True Negatives (TN)	4070	4058
False Positives (FP)	74	86
False Negatives (FN)	6	5
Brier Score	0.017	0.025
Sensitivity	0.84	0.87
Specificity	0.98	0.98
AUC	**0.91**	**0.92**

Supplementary Table 10. Preliminary and updated model results for known *PRKN* CNVs in held-out CORIELL PD Cases. Visually-confirmed samples with predicted values above 0.8 were fed back into the updated model's training set to create the training sets for our final models.

	***PRKN* Deletions**	***PRKN* Duplications**	***SNCA* Duplications***
True Positives (TP)	26	6	7
True Negatives (TN)	1201	1226	1188
False Positives (FP)	5	0	39
False Negatives (FN)	2	2	0
Brier Score	0.036	0.003	0.027
Sensitivity	0.929	0.750	1.000
Specificity	0.996	1.000	0.968
AUC	0.979	0.826	0.996
AUC for Visible Samples	**1.000**	**0.922**	**0.996**

Supplementary Table 11. Summary of model predictions on combined MLPA data from PFP and STELLENBOS cohorts. Asterisks denotes that the final duplication model was used to make predictions on *SNCA;* however, both duplications and triplications were identified.

Long-read WGS

	**Deletions**		**Duplications**	
	PRKN	***LINGO2***	***PRKN***	***MAPT***
True Positives (TP)	6	0	0	3
False Positives (FP)	0	0	0	0
PPV	**1**	**-**	**-**	**1**

Short-read WGS

	**Deletions**		**Duplications**	
	PRKN	***LINGO2***	***PRKN***	***MAPT***
Total True Positives (TP)	16	4	3	53
Total False Positives (FP)	2	0	0	14
Average PPV	**0.95**		**0.90**	

Supplementary Table 12. Summary of Positive Predictive Values (PPV) for final model predictions above 0.9 on PPMI long-read WGS data and predictions above 0.5 on short-read WGS data.
