# Supplementary material for: CNV-Finder: Streamlining Copy Number Variation Discovery
Source: bioRxiv. 2024 Nov 23:2024.11.22.624040. Preprint. [Version 1] doi: 10.1101/2024.11.22.624040 (PMC11601614; doi:10.1101/2024.11.22.624040)
Supplement: Supplementary file 1 [file media-1.pdf]

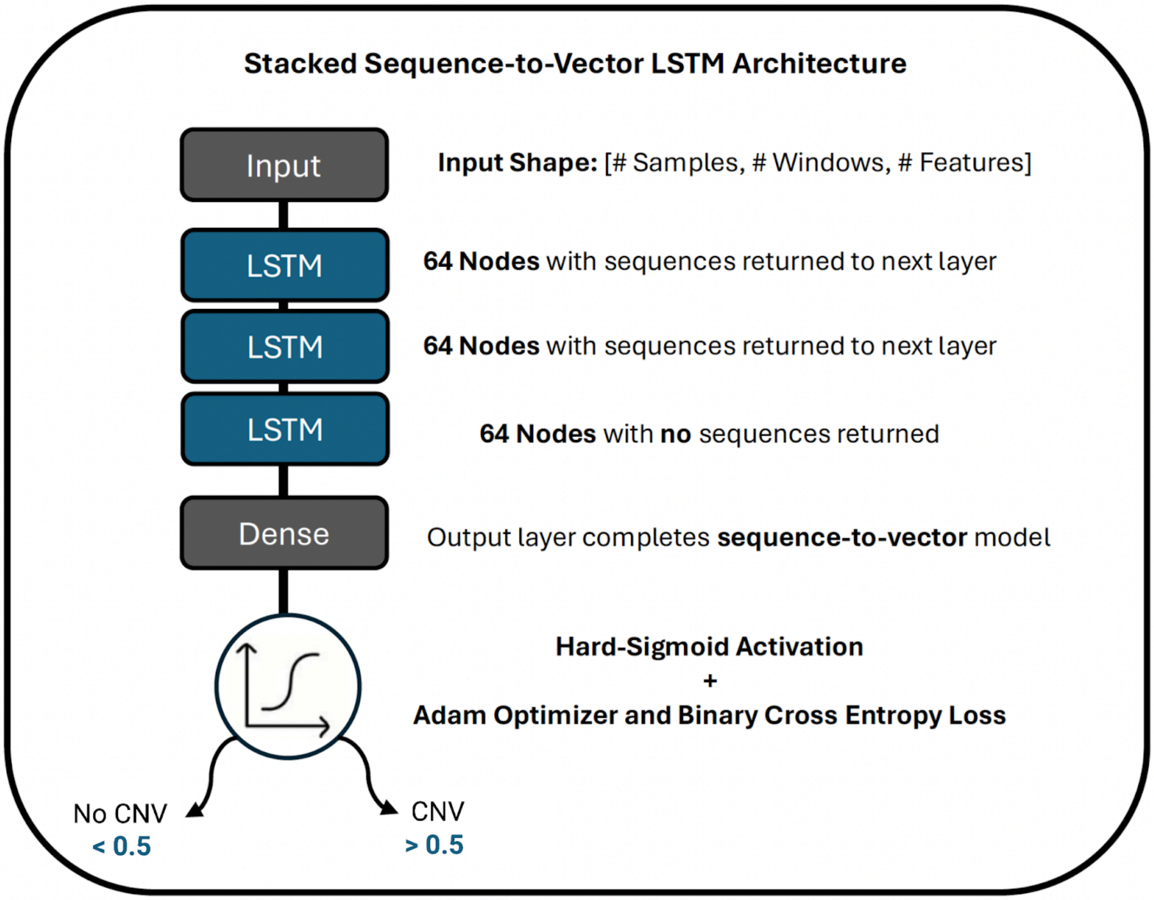

Supplementary Figure 1. Overview of Long Short-Term Memory model architecture.

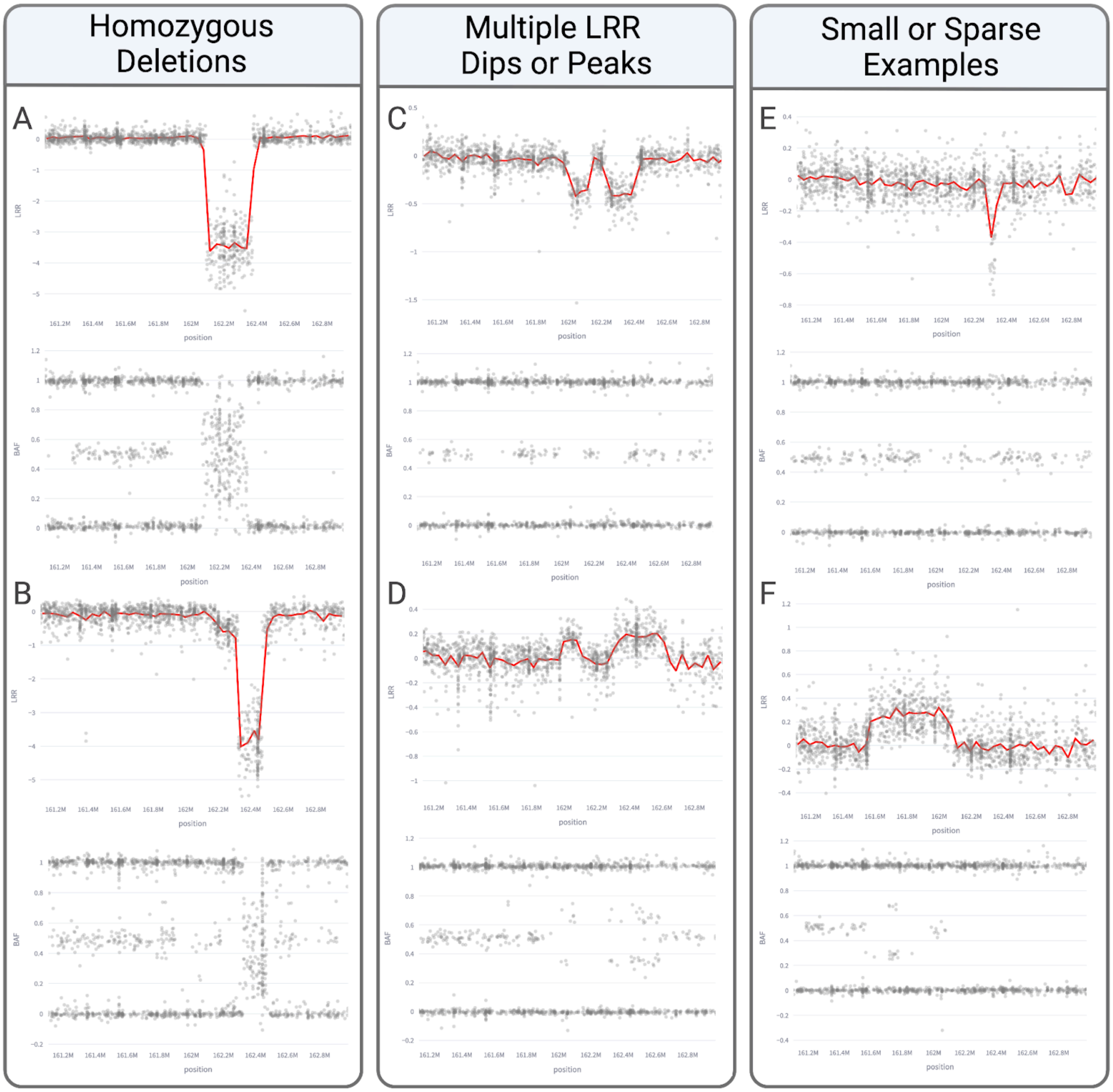

Supplementary Figure 2. Atypical samples identified by models without explicit training of these categories.

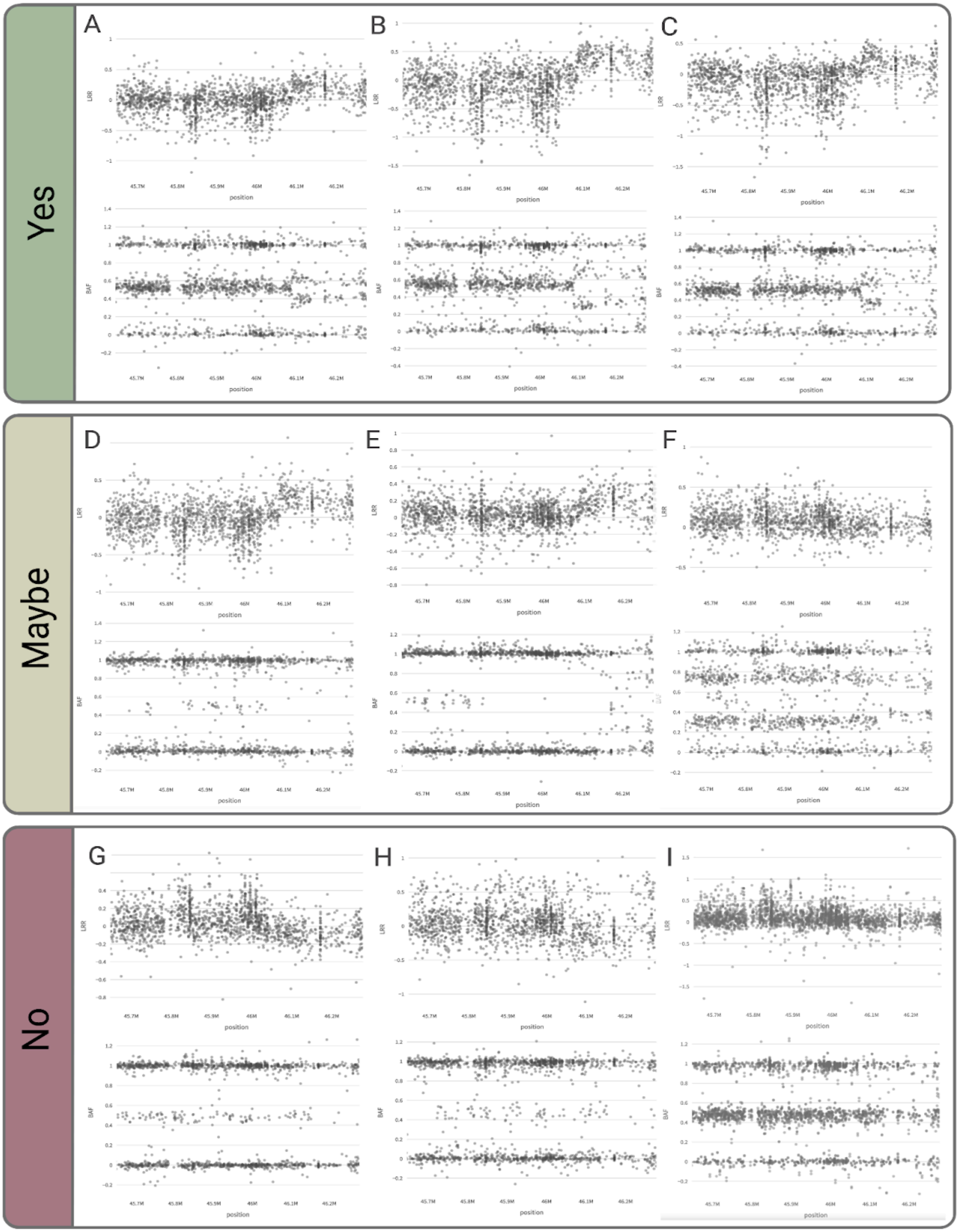

Supplementary Figure 3. Examples of classification used on duplication near *MAPT* during visual confirmation.
